# Adsorption of Imidazolium-Based ILs Combined on Activated Carbon Obtained from Grape Seeds

**DOI:** 10.3390/molecules30234595

**Published:** 2025-11-29

**Authors:** Ismael F. Mena, Elena Diaz, Jose Palomar, Angel F. Mohedano

**Affiliations:** 1 Chemical Engineering Department, Universidad Autónoma de Madrid, Campus de Cantoblanco, 28049 Madrid, Spain; ismael.fernandez@urjc.es (I.F.M.); elena.diaz@uam.es (E.D.); pepe.palomar@uam.es (J.P.); 2 Chemical and Environmental Engineering Group, Escuela Superior de Ciencias Experimentales y Tecnología (ESCET), Universidad Rey Juan Carlos, c/Tulipán s/n, 28933 Móstoles, Spain; 3 Institute for Advanced Research in Chemistry, Universidad Autónoma de Madrid, 28049 Madrid, Spain

**Keywords:** ionic liquids, adsorption, activated carbon, grape seeds, NTf_2_ anion

## Abstract

In this work, the adsorption of imidazolium-based ionic liquids containing the bis(trifluoromethanesulfonyl) imide anion (NTf_2_^−^) from aqueous phase was evaluated using different activated carbons (ACs). Three commercial Acs and two Acs prepared from grape seeds (one produced by pyrolysis and the other by hydrothermal carbonization (HTC), both activated with potassium hydroxide) were tested, assessing the adsorption of both the cation and the anion. For commercial ACs, similar adsorption performances were observed, with maximum adsorption capacities ranging from 0.85 to 1.08 mmol g^−1^. These values increased under acidic conditions (pH 4), reaching 1.74 mmol g^−1^ for the 1-butyl-3-methylimidazolium cation (Bmim^+^) and 1.87 mmol g^−1^ for NTf_2_^−^. Among the prepared ACs, the HTC-derived AC showed slightly higher capacities than the commercial samples, while the pyrolysis-derived AC exhibited the highest adsorption capacity for BmimNTf_2_ (3.36 mmol g^−1^ at pH 4). In terms of reusability, the pyrolysis-derived AC maintained 84% of its initial adsorption capacity between the third and fifth regeneration cycles. These results highlight the high adsorption performance and recyclability of grape-seed-derived activated carbons, demonstrating their potential for the removal of ionic liquids from aqueous environments.

## 1. Introduction

Ionic liquids are salts with a melting point below 100 °C formed by an organic cation and an organic or inorganic anion [1]. In recent decades, ILs have been considered novel solvents due to their remarkable properties. Among them, their low vapor pressure and their chemical and thermal stability have allowed them to be considered as “green solvents” [2]. However, depending on the hydrophobicity of the combined cations and anions, some ILs can present relatively high solubility in water [3]. Consequently, they can appear in wastewater because of their synthesis procedure or due to their use. Several authors have evaluated the ecotoxicity and biodegradability of various IL families combined with different anions, questioning their consideration as “green solvents” [4,5,6,7]. Imidazolium, pyridinium, and pyrrolidinium families present relatively high toxicity with respect to ammonium or phosphonium ones, and present low biodegradability [8,9]. Moreover, the use of a fluorinated anion like the hexafluoroborate (PF_6_^–^) or the bis(trifluoromethanesulfonimide) (NTf_2_^–^) produces an increase in the IL toxicity respect to halides or organic acids due to the possible hydrolysis to hydrofluoric acid [10], and present low biodegradability being able to permeate on the cell membranes, making them a problem to the aquatic environmental [11]. Therefore, there is a need to assess new processes aimed at the removal of ionic liquids (ILs), especially those exhibiting higher ecotoxicity, such as imidazolium-based ILs and those with the NTf_2_ anion.

Different advanced oxidation processes (AOPs) have been evaluated as destructive treatments for ILs wastewater [12]. Among them, photocatalysis [13,14], Fenton [15], catalytic wet peroxide oxidation [16], electrolysis [17,18], and electroFenton [19,20] have been tested satisfactorily, achieving high removal rates in terms of cation and mineralization. Regarding the behavior of the anion, Fenton reagent can degrade aromatic anions, such as tosylate [21], and electrolysis can degrade organic anions like acetate [22]. Specifically, the NTf_2_ anion is recognized as a refractory compound in various AOPs, since hydroxyl radicals are unable to break the C–S, S–N, and C–F bonds [23].

As an alternative to AOPs, the adsorption of ILs from the aqueous phase has emerged as a promising strategy for wastewater treatment. The main advantage of this approach lies in the possibility of recovering and reusing the ILs without altering their chemical structure. This is particularly relevant given the high economic value and versatility of ILs in various industrial applications [24,25]. Adsorption has been extensively studied as an effective method for the removal of various contaminants from aqueous media, with activated carbon consistently highlighted as the preferred adsorbent due to its high surface area and exceptional adsorption capacity. This widespread applicability is reflected in the global market trends: the activated carbon market was valued at USD 4.4 billion in 2023 and is projected to reach USD 8.4 billion in 2030, according to a study published by Markets and Markets in 2024 [26]. Moreover, water treatment applications reached more than 40% of the global AC consumption in 2023, according to a study published by Grand View Research in 2024 [27]. Different raw materials have been used to obtain ACs, such as wood, peat, or petroleum pitch, among others. The large number of agricultural wastes generated poses a great challenge to produce carbon materials by means of pyrolysis and hydrothermal carbonization (HTC) [28]. Pyrolysis of biomass wastes are carried out in two steps: drying of the raw material and carbonization under N_2_ atmosphere at high temperature. Nevertheless, HTC emerges as an alternative for obtaining a solid product called hydrochar, which presents higher carbon content than the raw material at relatively mild conditions in the presence of water compared with pyrolysis (180–300 °C and autogenous pressure) [29]. In general, an activation process is needed after pyrolysis and HTC processes to achieve a high surface area and pore volumes. Different reagents can be used in the chemical activation process, such as KOH or K_2_CO_3_ [30], phosphoric acid [31], or FeCl_3_ [32,33]. Different agricultural wastes have been used in this sense, such as olive [34,35,36], rice [37,38], or pomegranate waste [39]. Specifically, grape seeds represent up to 15% of the wine industry waste, and they have been used as precursors of different carbon materials by means of pyrolysis [31,40] or HTC [29,41], obtaining high adsorption capacities of herbicide and pharmaceutical pollutants.

Regarding the adsorption of ILs, Table 1 shows some results focused on the study of the IL composition to know the influence of the cation family, the cation alkyl side chain, the characteristics of the anion on the adsorption capacity, or the influence of operation conditions such as temperature or pH. Moreover, the use of different adsorbents has been evaluated, using different commercial ACs, with or without thermal or oxidation treatment. Furthermore, some studies evaluated the adsorption capacity of ACs synthesized by different biomass wastes, such as wood, artichokes, or peanut shells, among others. Table 1 also summarizes the properties of the activated carbon, such as the BET area, pH_slurry_/pH_ZPC_, and micropore volume, which are evaluated to determine the best characteristic for a good IL adsorbent. In general, these works have related the hydrophobicity of the cations and anions with the cation adsorption capacity, obtaining better results when the temperature was reduced. Moreover, when the pH increased, the adsorption capacity increased, due to the high affinity between the cation and the negatively charged surface of the AC at high pHs. In terms of the ACs, the increase in the BET area and the micropore volume produces a higher cation adsorption capacity of the ILs. Although activated carbons present a high adsorption capacity for ILs, there is a lack of knowledge to ensure that the adsorption of the cation and the anion of the ILs follows the same pattern.

The main objective of this study is to explore the potential of novel activated carbons derived from agricultural waste, specifically grape seeds, as sustainable precursors. Two types of carbonaceous materials were synthesized via pyrolysis and HTC, followed by chemical activation with KOH. This approach seeks to valorize agro-industrial waste into high-performance porous materials for advanced environmental applications, particularly for the removal of ILs from aqueous media. Seven imidazolium-based ILs were selected to investigate the influence of both the anion type and the alkyl side-chain length on the adsorption behavior. The adsorption capacity of the prepared materials was evaluated separately for the cation and the anion and correlated with their chemical composition and textural properties. Furthermore, adsorption–desorption cycles were carried out to assess the reusability and stability of the most effective adsorbent in terms of adsorption capacity, with the ultimate goal of identifying an efficient and sustainable material for IL removal.

## 2. Results and Discussion

### 2.1. Characterization of the Activated Carbons

**Figure 1:**
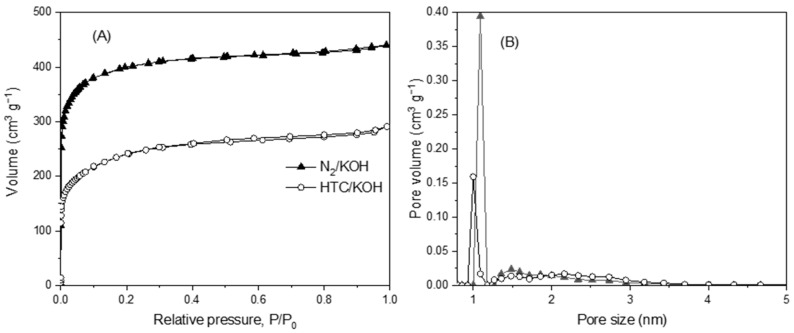
Nitrogen adsorption/desorption isotherms (**A**) and pore size distribution (**B**) for the materials prepared by activation (N_2_/KOH) and hydrothermal carbonization (HTC/KOH).

Table 2 shows the main properties of the commercial and prepared ACs. As shown, all the ACs exhibit BET surface areas between 800 and 1400 m^2^ g^−1^, with Chem and N_2_/KOH achieving the highest values. Regarding to the porosity, GXS and Chem stand out by a high mesoporosity, with mesopore volumes of 0.170 and 0.270 cm^3^ g^–1^, respectively (4–10 times higher than the other ACs), whereas the Merck AC and those synthetized from grape seeds are mainly microporous materials. Figure 1 presents the N_2_ adsorption–desorption isotherms (A) and the pore size distribution (B) of the materials synthesized in this study: pyrolysis (N_2_/KOH) and hydrothermal carbonization (HTC/KOH) of grape seeds, followed by KOH activation. Further details regarding the characterization of the commercial activated carbons are available in García-Delgado et al. (2018) [53]. Lemus et al. (2012) [24] evidenced that the adsorption of the IL OmimPF_6_ are clearly related to the volume of pores up to 8 nm (micropore and narrow mesopore). In this sense, Chem presents the highest amount of pore volumes up to 8 nm, followed by GXS and N_2_/KOH, with HTC/KOH being the material with the lowest accumulation of micro- and mesoporosity. Regarding the elemental analysis, it can be appreciated that commercial ACs present similar characteristics, with the carbon content between 84 and 90 wt. %. Moreover, GXS presents almost double the ashes content than Merk and Chem (8.4 wt. % against 4.7 and 4.8, respectively). The carbon content of AC synthesized from grape seeds is 15–20 wt. % higher than the raw material (between 70–75 wt. %), although lower than that exhibited by the commercial materials. Additionally, these ACs present a high number of ashes, around 15 wt. %, related to the residual ash derived from the grape seed precursor and/or from the KOH activation process. Concerning the commercial ACs pH_slurry_, Merck presented a pH_slurry_ close to neutrality, and GXS and Chem had an acidic pH_slurry,_ whereas the chemical activation with KOH produced basic activated carbons. Finally, the TPD results show that Chem presented the highest amount of SOG, while the treatment of the grape seed produced a generation of SOGs similar to that shown by the commercial ACs.

### 2.2. Adsorption of Bmim-Based ILs

**Figure 2:**
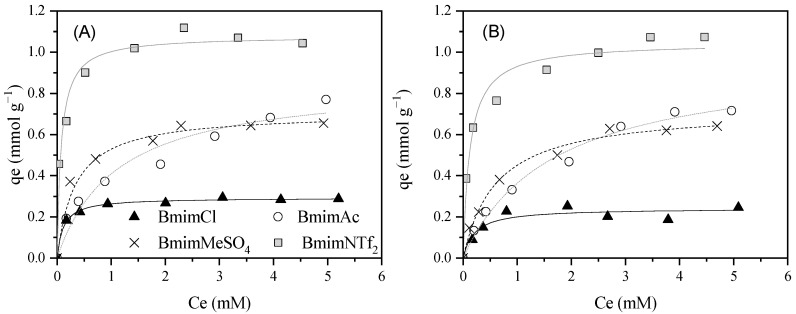
Experimental data (dots) and Langmuir fits (curves) for Bmim^+^ (**A**) and anion (**B**) adsorption equilibrium isotherms at 20 °C and neutral pH (see Table 3). [Merck] = 0.5 g L^−1^, [IL] = 0.25–5 mM, 200 rpm.

Isotherm adsorption of the cation and anion of different Bmim-based ILs was evaluated to study, on one hand, if there is an influence of the type of anion on the adsorption, and on the other hand, to elucidate if the ILs’ adsorption is governed by the adsorption of the ion pair or the cation/anion separately. Figure 2 shows the results obtained in the adsorption experiments of BmimCl, BmimAc, BmimMeSO_4_, and BmimNTf_2_ using Merck. In all the cases, the Bmim^+^ adsorption isotherm is like its corresponding anion, making evident that the adsorption phenomenon takes place via an ion pair. In general, other authors have measured the cation concentration considering that the cation adsorption is similar to IL adsorption, and consequently, the anion adsorption [42,45,47,49,54]. By comparing the adsorption behavior of the different ILs with respect to their anionic constituent at comparable neutral pH values (see Table 3), a correlation can be established between this parameter and the hydrophobicity of the ILs. According to COSMO-RS calculations, the following trend was obtained based on the logarithm of the octanol–water partition coefficient (Log P): BmimCl (−2.92) < BmimMeSO_4_ (−2.07) < BmimAc (−0.040) < BmimNTf_2_ (3.44). BmimCl, being the most hydrophilic ionic liquid, exhibited the lowest adsorption capacity, whereas BmimNTf_2_ showed the highest adsorption capacity due to its pronounced hydrophobicity. Interestingly, at concentrations between 0.25 and 3 mM, the adsorption of BmimAc was lower than that of BmimMeSO_4_, despite the latter being less hydrophobic. However, as the IL concentration increased, BmimAc adsorption surpassed that of BmimMeSO_4_, whose adsorption remained nearly constant.

BmimNTf_2_ presents a higher ecotoxicity value than the other ILs [11,55], due to the high hydrophobicity of NTf_2_^−^ compare with acetate or chloride as an anion, combined with a null biodegradability using an activated sludge [11]. Additionally, previous works established NTf_2_^−^ as a recalcitrant compound in different advanced oxidation processes, such as electrolysis or catalytic wet peroxide oxidation, whereas the imidazolium cation or organic anions like acetate are degraded. For these reasons, the adsorption of ILs using NTf_2_^−^ can be regarded as a promising alternative for their removal from water. Consequently, the subsequent research focused on investigating the adsorption of ILs with NTf_2_^−^.

**Figure 3:**
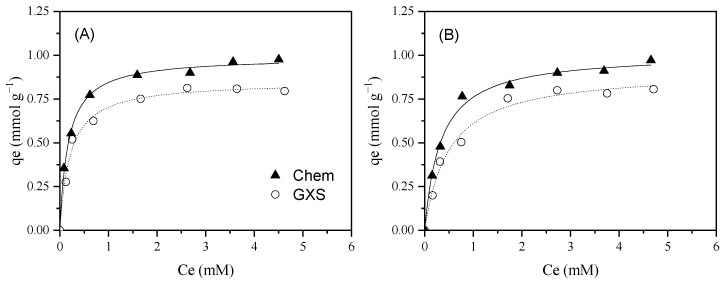
Experimental data (dots) and Langmuir fits (curves) for Bmim^+^ (**A**) and NTf_2_^−^ (**B**) adsorption equilibrium isotherms at 20 °C and neutral pH (see Table 3) on different commercial activated carbons. [AC] = 0.5 g L^−1^, [IL] = 0.25–5 mM, 200 rpm.

The adsorption of BmimNTf_2_, the most toxic ionic liquid studied and the one exhibiting the highest adsorption capacity (Figure 2), was investigated using other commercial ACs. As can be seen in the comparison between Figure 2 and Figure 3, the adsorption capacity of commercial ACs follows the following trend in the BmimNTf_2_ adsorption: Merck > Chem > GXS. The analysis of ACs characterization (Table 2) reveals that BmimNTf_2_ adsorption was neither related to the A_BET_ nor the micropore volume nor to the amount of SOGs [51]. However, the pH_slurry_ seems to have a relevant influence on the adsorption capacity. When the AC presents a lower pH_slurry_ than the reaction medium, the material surface is negatively charged and favors the cation adsorption, whereas a higher pH_slurry_ value favors the anion adsorption [56]. In these cases, the use of Merck, an AC with neutral pH_slurry_, produced high adsorption capacity, while when the pH_slurry_ was reduced, the adsorption capacity was lower, as occurs with Chem or GXS. This fact indicates that the anion component had a key role in the adsorption of the BmimNTf_2_ due to NTf_2_^−^ adsorption was favored in ACs with a neutral or positively charged surface, because of the electrostatic attraction forces. Moreover, comparing Chem and GXS, both present negatively charged surfaces at the experimental pH; therefore, the higher BET area and micropore volume of Chem appear to be the decisive factors explaining its higher adsorption capacity relative to GXS.

**Figure 4:**
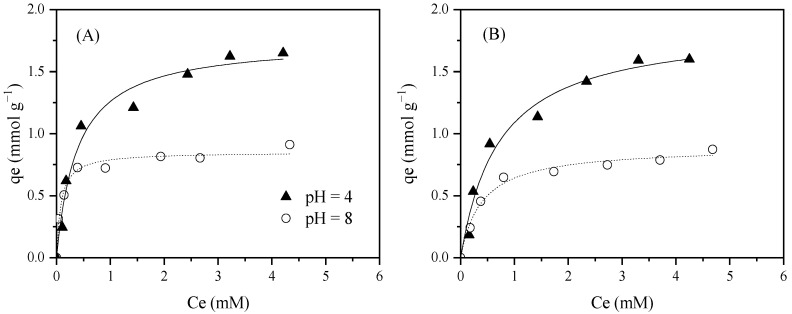
Experimental data (dots) and Langmuir fits (curves) for Bmim^+^ (**A**) and NTf_2_^−^ (**B**) adsorption equilibrium isotherms at 20 °C at different pH. [Merck] = 0.5 g L^−1^, [IL] = 0.25–5 mM, 200 rpm.

The influence of medium pH on the adsorption phenomenon has been studied using different ILs, such as BmimCl, OmimCl, OpyBr, or EmimBF_4_. In general, these hydrophilic ILs present a higher adsorption capacity at basic pH. This is related to the low affinity between the Bmim^+^ and the carbon material at acid pH, due to the repelling charges between the cation and the positively charged AC [43,47,49,52]. The role of pH was examined using the activated carbon that showed the highest adsorption capacity (Merck) and BmimNTf_2_ as the adsorbate. As can be seen in Figure 4, the BmimNTf_2_ maximum adsorption capacity took place at a pH value of 4, provoking an increase in the medium pH and a decrease in the adsorption. In this case, the NTf_2_^−^ presents a high hydrophobic behavior and, because it is an anion, its adsorption can be favored by ACs with a positively charged surface. Due to Merck presenting a pH_slurry_ of 7.7, using a pH of 4 in the adsorption medium, the material surface charged positively, favoring the anion adsorption. For that reason, the BmimNTf_2_ adsorption is higher at acidic conditions in contrast with other ILs with hydrophilic anions. However, when the medium pH was adjusted to 8, the adsorption capacity of the material was reduced because the Merck surface was slightly negatively charged, producing repelling electrostatic forces with NTf_2_^−^.

**Figure 5:**
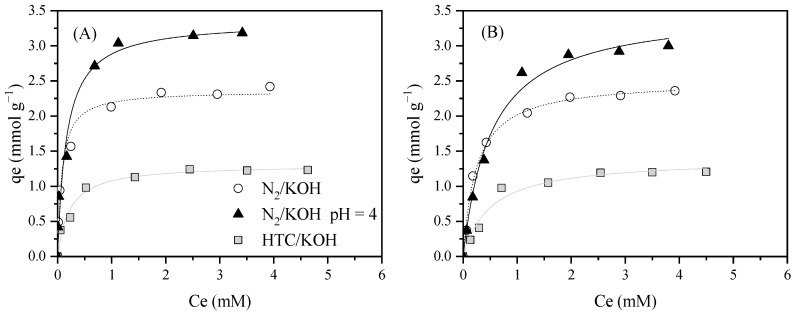
Experimental data (dots) and Langmuir fits (curves) for Bmim^+^ (**A**) and NTf_2_^−^ (**B**) adsorption equilibrium isotherms at 20 °C and neutral pH (see Table 3) and pH = 4 using grape-seed-based ACs. [AC] = 0.5 g L^−1^, [IL] = 0.25–5 mM, 200 rpm.

Finally, the adsorption study of BmimNTf_2_ was extended to the ACs synthesized from grape seeds. As can be seen in Figure 5, cation and anion adsorption isotherms were quite similar, as occurs with the commercial ACs, making evident the adsorption of both components together. Comparing the adsorption capacity using the highest IL concentration, N_2_/KOH presented a value 1.96 times higher than HTC/KOH. This effect can be attributed to the higher BET area and micropore volume developed in N_2_/KOH. However, HTC/KOH presented a slightly higher adsorption capacity than Merck (1.23 mmol g^−1^ against 1.04 mmol g^−1^ for HTC/KOH and Merck, respectively). In this case, although both materials exhibited similar textural properties, the pH_slurry_ of HTC/KOH caused the AC surface to become positively charged, favoring the NTf_2_^−^ adsorption, and consequently, the IL adsorption. In contrast, Merck presented a pH slurry similar to an IL solution, showing that the material displayed a neutral surface. An additional experiment was carried out using the N_2_/KOH at an acidic pH. As can be seen, the adsorption capacity at acidic pH is 1.4 times superior to that obtained at neutral pH. This fact can be associated with the highly positively charged surface that this material presented at pH 4, favoring the anion interaction with the AC.

**Figure 6:**
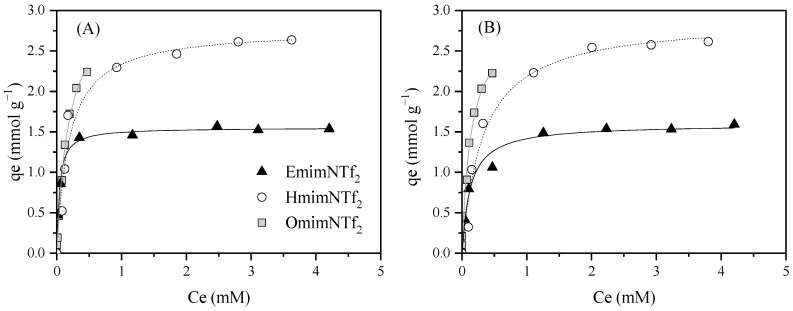
Experimental data (dots) and Langmuir fits (curves) for Cation (**A**) and NTf_2_^−^ (**B**) adsorption equilibrium isotherms at 20 °C and neutral pH of different imidazolium IL using N_2_/KOH. [N_2_/KOH] = 0.5 g L^−1^, [IL] = 0.25–5 mM except for OmimNTf_2_ (0–1 mM), 200 rpm.

Once it was established that the activated carbon N_2_/KOH exhibited the highest adsorption capacity for BmimNTf_2_, the influence of the alkyl side chain on ionic liquid adsorption was evaluated. Other authors, such as Ushiki et al. (2017) [50] or Zhang et al. (2018a) [51], evidenced an increase in the adsorption capacity when the cation alkyl side chain increased. Both attributed this result to an increase in IL hydrophobicity when the alkyl side chain increased from 4 to 8 C atoms. Figure 6 shows the length of the cation influence in the IL adsorption at neutral pH with N_2_/KOH. Adsorption capacity follows the order OmimNTf_2_ > HmimNTf_2_ > BmimNTf_2_ > EmimNTf_2_, as was previously reported. When the alkyl side chain increased from two to eight carbon atoms, the hydrophobicity, and consequently the adsorption capacity onto the AC, increased.

**Figure 7:**
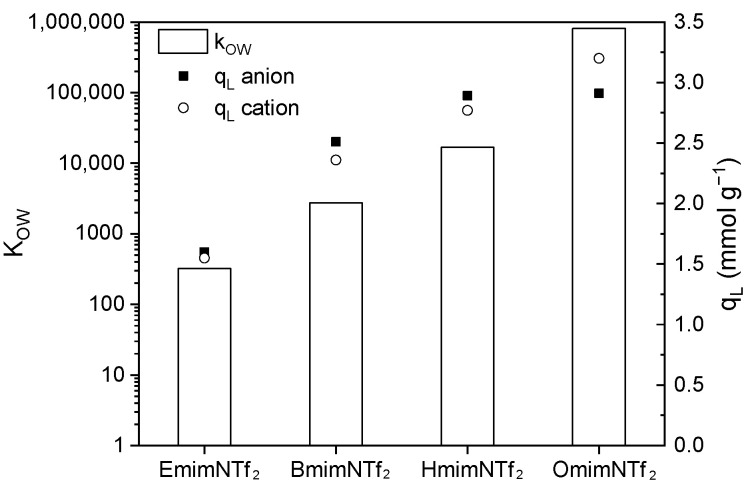
K_OW_ coefficient estimated by COSMO-RS and cation/anion adsorption capacity of EmimNTf_2_, BmimNTf_2_, HmimNTf_2_, and OmimNTf_2_. [N_2_/KOH] = 0.5 g L^−1^, T = 20 °C, 200 rpm, neutral pH.

Table 3 collects the results of the Langmuir parameters. Langmuir equation reproduces the experimental values well, with a determination coefficient (r^2^) superior to 0.946, except for the chloride anion in the BmimCl adsorption (r^2^ = 0.882). As can be seen, the parameter q_L_, which represents the maximum adsorption capacity, is similar for anion and cation adsorption in all the cases (q_L_ cation/q_L_ anion close to 1). Comparing these parameters with those obtained in the literature (Table 1), the maximum adsorption capacity of the ACs studied showed similar values to those previously obtained. Focusing on the ACs synthesized from grape seeds, N_2_/KOH is the material that presents the highest IL adsorption capacity, achieving the highest q_L_ value of the studied (q_L_ = 3.53 mmol g^−1^) in the BmimNTf_2_ adsorption at pH = 4. This value is similar to that obtained by Lemus et al. (2012) [24] in the OmimPF_6_ adsorption by the Capsuper commercial AC (q_L_ = 3.5 mmol g^−1^) and that obtained by Zhang et al. (2018a) [51] in the DcmimCl adsorption using an ordered microporous carbon ZTC (q_L_ = 3.4 mmol g^−1^). In these cases, the IL cation studied presents a longer alkyl side chain compared with the Bmim^+^, favoring the IL adsorption for the hydrophobicity associated with those cations. However, the anion studied in this work presents a higher hydrophobicity than the chloride, making the adsorption capacity similar in both cases. The activated carbon synthesized in this study exhibited a higher adsorption capacity than the commercial samples, clearly demonstrating the competitive performance of activated carbon produced from waste materials. The K_OW_ coefficients of the ILs studied in this work were estimated using COSMO-RS. As shown in Table 3, the K_OW_ values, and consequently the hydrophobicity, increased for the ILs containing the NTf_2_ anion. In fact, the K_OW_ value of BmimNTf_2_ is more than 3000 times higher than that of BmimAc, and it further increases with the length of the alkyl side chain of the cation (approximately 250 times higher when the chain length increases from 2 to 10 carbon atoms). Figure 7 shows the K_OW_ coefficients of the ILs containing the NTf_2_ anion. A clear correlation can be established between the K_OW_ values and the adsorption capacities (q_L_), as expected.

**Figure 8:**
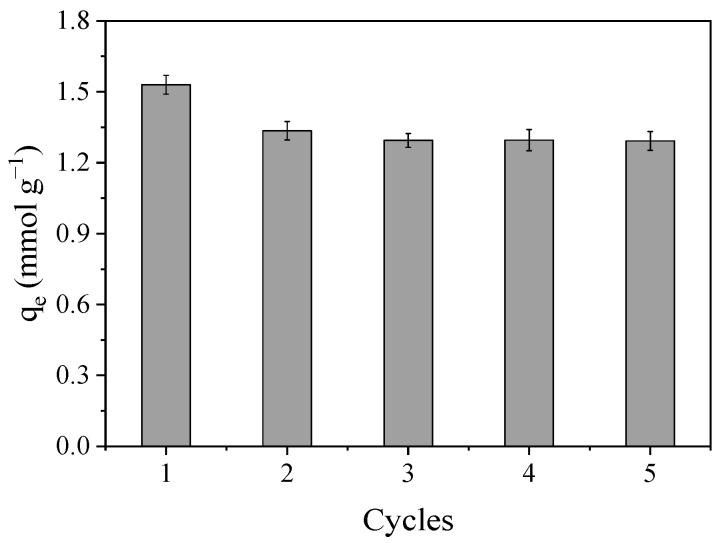
Bmim^+^ adsorption capacity along five consecutive cycles. [N_2_/KOH] = 0.5 g L^−1^, [BmimNTf_2_] = 1 mM, T = 20 °C, 200 rpm, neutral pH.

Finally, the reusability of the N_2_/KOH has been evaluated in 5 cycles of adsorption–desorption of 1 mM of BmimNTf_2_ at neutral pH using acetone as regenerating agent. The high cost of ILs and the environmental risk associated with them make their recovery a critical point. Figure 8 shows the mean adsorption capacity of N_2_/KOH in each cycle. As can be seen, the Bmim^+^ adsorption capacity was reduced from 1.53 mmol g^−1^ to 1.29 mmol g^−1^ from the first to the third reuse cycle, and then it remained constant. The material reaches an adsorption capacity of 84% with respect to the initial one. This value is in concordance with others obtained in the literature. Lemus et al. (2012) [24] determined, using Merck and Capsuper commercial ACs and acetone as regenerating agent, that the adsorbent maintained a 90% of the initial adsorption capacity after three cycles, whereas Shi et al. (2016) [47] obtained a 82 and 88% of the initial adsorption capacity after 10 cycles of adsorption–desorption in a straw and wood based biochar, respectively, and a HCl solution (pH = 0.5) as regenerating agent. Therefore, the activated carbon synthesized from the pyrolysis of grape seeds presented high adsorption capacity and reusability in ILs adsorption.

## 3. Materials and Methods

### 3.1. Ionic Liquids

The ILs studied in the adsorption experiments were purchased from Sigma Aldrich (Darmstadt, Deutschland) and Iolitec (Heilbronn, Deutschland) and are presented in Table 4. Acetone (>99.5% purity) was provided by SCHARLAB (Barcelona, Spain), and potassium hydroxide (KOH, 85% purity) was provided by PanReac AppliChem (San Fernando de Henares, Spain).

### 3.2. Activated Carbon: Preparation and Characterization

Three commercial activated carbons (ACs) were used in adsorption experiments: Chemviron (Chem, supplied by Norit (Amersfoort, The Netherlands)), GXS (supplied by Norit), and Merck (supplied by Merck (Rahway, NJ, USA)). The ACs were crushed and sieved below 100 µm.

Moreover, grape seeds of red wine variety “Tinta de Toro” from Toro (Zamora, Spain) were used as precursor for two activated carbons. As pretreatment, the seeds were washed with Milli-Q water and dried overnight at 105 °C. Grape seeds are characterized by an absence of porosity and a relatively high carbon (≈55%) and low ash (close to3%) content. One of the ACs (N_2_/KOH) was obtained by pyrolysis of the grape seeds in a rotatory quartz furnace (CARBOLITE CB HTR 11/150P8) at 600 °C, reached at 10 °C min^−1^, for 2 h using a continuous flow of 1 NL min^−1^ of N_2_, whereas the AC (HTC/KOH) was obtained by HTC of 20 g of the dried grape seeds at 220 °C for 16 h in a 250 mL stainless steel reactor with a ratio water to grape seeds of 40 wt. %. Both materials were subjected to an activation process with KOH using a char: KOH mass ratio of 1:3. After that, the char and the KOH were crushed, mixed, and subjected to heating at 750 °C (ramping up 10 °C min^−1^) for 1 h under N_2_ atmosphere (100 mL N min^−1^) in a Nabertherm Series R tubular furnace. Before their use, the ACs were washed with a HCl solution (5 M) and progressively with Milli-Q water until constant pH. Finally, they were dried at 105 °C for 24 h and crushed and sieved below 100 µm.

Nitrogen adsorption–desorption isotherms at 77 K were carried out to obtain the porous structure of adsorbents using a Micromeritics Tristar 3020 automated volumetric gas adsorption instrument (Micromeritics Instrument Corporation, Gwinnett County, Georgia). A Micromeritics VacPrep 061 degassing system was used previously to degasify under vacuum samples (0.15 g) at 150 °C for 7 h. The BET equation was used to obtain the surface area (A_BET_), and micropore volume was calculated using the t-method and total volume of mesopores (up to 8 nm) from the difference between N_2_ adsorbed at 0.95 relative pressure and the micropore volume [24]. Elemental analysis (C, H, N, and S) was quantified with a LECO CHNS–932 elemental analyzer. Ash content was determined according to ASTM D1506–99 method, and the oxygen content in mass was calculated by difference from 100%. The pH_slurry_ was determined by measuring the pH of an aqueous suspension of carbon (1 g) in Milli-Q water (10 mL) [57]. Temperature-programmed desorption under N_2_ flow (1 mLN min^−1^) (TPD–N_2_) was used to analyze the amount of surface oxygen groups (SOG) presented in the carbon materials. A sample of 0.1 g was introduced in a vertical quartz reactor and heated from room temperature to 900 °C using a heating rate of 10 °C min^−1^. The amount of CO and CO_2_ released was continuously measured by a Siemens Ultramat 23 NDIR analyzer (Siemens, Munich, Germany).

### 3.3. Adsorption–Desorption Experiments

Equilibrium adsorption tests were carried out in stoppered glass bottles (100 mL) placed in an orbital incubator (Comecta Orbital Shaking Incubator (Glassco Scientific & Analytical Company, Tikatuli, Bangladesh), model D–1102) at 200 rpm equivalent stirring rate and 20 °C. ILs solutions were prepared with concentrations from 0.25 to 5 mmol L^−1^ in Milli-Q water, except for OmimNTf_2_, whose concentration was between 0.1 and 1 mmol L^–1^ due to its low solubility. An amount of 25 mg of activated carbon was put in contact with 50 mL of IL solution, corresponding to an AC concentration of 0.5 g L^−1^. The equilibrium time was 24 h for all the samples. The reusability of the best adsorbent was evaluated in 5 consecutive cycles of IL adsorption–desorption. Desorption was carried out suspending the AC in acetone in a concentration of 500 mg L^−1^ under continuous stirring for 1.5 h. After that, the AC was separated by filtration, dried in an oven at 70 °C for 16 h, and used in the following experiment.

Imidazolium cation concentration was determined by means of a high-performance liquid chromatograph (Varian Prostar 240) with an UV–Vis detector (Varian Prostar 325 (SpectraLab Scientific Inc., Markham, ON, Canada)) operated at 218 nm. A Synergy 4mm Phenomenex Polar–RP 80 A column 15 cm long × 4.6 mm was used as stationary phase. A mixture of phosphate buffer and acetonitrile (from 95:5 to 60:40% *v*/*v* depending on the cation analyzed) was pumped at 0.75 mL min^−1^, and the injection volume used was 100 µL.

Chloride, acetate, and methylsulfate anions were analyzed by means of ion chromatograph with chemical suppression (DIONEX ICS–900) (Thermo Fisher Scientific Inc., Waltham, MA, USA) equipped with a Dionex IonPac AS22 4 × 250 mm column. The mobile phase was a mixture of 1.4 mM NaHCO_3_ and 4.5 mM Na_2_CO_3_ at 1 mL min^−1^. TOC was determined with a Shimadzu TOC–VCSH TOC analyzer. NTf_2_^−^ concentration was determined by difference from TOC total and TOC associated to the cation concentration.

The equilibrium data were fitted by the Langmuir isotherm equation, described as

$$q_{e}=\frac{q_{L}\cdot k_{L}{\cdot C}_{e}}{1+k_{L}{\cdot C}_{e}}$$

being *q_e_* the equilibrium concentration of adsorbate in solid phase (mmol g^–1^), *C_e_* equilibrium concentration of adsorbate in fluid phase (mmol L^–1^) and *q_L_* (mmol g^–1^) and *k_L_* (L mmol^–1^) the empirical coefficients in Langmuir equation.

## 4. Conclusions

Activated carbons synthesized from grape seeds through pyrolysis and hydrothermal carbonization exhibited outstanding performance in the adsorption of imidazolium-based ionic liquids (ILs), surpassing even commercial activated carbons. This study demonstrates the high efficiency of these sustainable carbonaceous materials in removing toxic compounds, particularly imidazolium ILs containing the NTf_2_^−^ anion, known for its environmental persistence and low biodegradability. The adsorption capacity reached 1.05 mmol NTf_2_^−^ g^−1^ for BmimNTf_2_, compared with 0.97, 0.73, and 0.24 mmol g^−1^ for the acetate, methylsulfonate, and chloride anions, respectively.

The activated carbon produced via pyrolysis under a nitrogen atmosphere, followed by chemical activation with KOH, showed the highest adsorption performance, attributed to its large BET surface area, high pore volume, and positively charged surface at neutral pH. Among the ILs tested, OmimNTf_2_ achieved the greatest adsorption capacity (2.91–3.20 mmol g^−1^) due to its pronounced hydrophobicity and strong ion-pairing interactions. Furthermore, the activated carbon retained 84% of its initial adsorption capacity after five adsorption–desorption cycles, confirming its stability, reusability, and potential as a sustainable adsorbent for the removal of persistent ionic liquids from aqueous environments.

## Figures and Tables

**Table 1:** A compilation of ILs adsorption studies from aqueous phase.

ILs	Material	Treatment	AC Characteristics	Adsorption Tests	Adsorption Capacity (mmol g^–1^)	Significant Results/ Best Operating Conditions	Ref.
BmimBF_4_ BmimCl BmimMeSO_3_ BmimNTf_2_ BmimOTf BmimPF_6_ EmimNTf_2_ HmimCl HmimPF_6_ OmimBF_4_ OmimCl OmimPF_6_	Commercial AC (Merck)	No treatment, Thermal treatment (900 °C) Nitric acid oxidation (900 °C) Ammonium persulfate oxidations (900 °C)	A_BET_ = 494–932 m^2^ g^−1^	C_0_ = 0–5 mM W = 250 mg L^−1^ T = 25–55 °C Neutral pH	q_L_ = 0.14–1.3	q_L_ = 1.32 mmol_Omim+_ g^−1^ IL: OmimPF_6_ T: 35 °C AC: similar in all cases	Palomar et al., 2009 [42]
BmimCl OmimCl OpyBr	2 commercial ACs (China and Calgon) Artichokes	The commercial carbons were washed and dried (110 °C) Artichokes were activated using phosphoric acid	A_BET_ = 984–2106 m^2^ g^−1^ pH_PZC_ = 6–9.5	C_0_ = 0–20 mM W = 1000–2000 mg L^−1^ T = 20–55 °C pH = 2–9	q_e_ = 0.4–2.3	q_e_ = 2.3 mmol_Omim+_ g^−1^ IL: OmimCl pH: 9 AC: Artichokes AC (pH_PZC_ = 6; highest O content)	Farooq et al., 2012 [43]
EmimCl BmimCl HmimCl OmimCl DmimCl DdmimCl TdmimCl HdmimCl EmimBF_4_ BmimBF_4_ HmimBF_4_ OmimBF_4_ DmimBF_4_ DdmimBF_4_ EmimPF_6_ BmimPF_6_ HmimPF_6_ OmimPF_6_ DmimPF_6_ DdmimPF_6_ EmimNTf_2_ PrmimNTf_2_ BmimNTf_2_ HmimNTf_2_ BmimOTf BmimTFA BmimMeSO_3_	5 commercial ACs (CAPSUPER, SXPLUS, GXS, Merck and ENA250G), silica and alumina	No treatment, thermal treatment (900 °C) and nitric acid oxidation.	A_BET_ = 79–1915 m^2^ g^−1^ pH_Slurry_= 3.3–8.1	C_0_ = 0–5 mM W = 250 mg L^−1^ T = 35 °C Neutral pH	q_L_ = 0.05–3.5	q_L_ = 3.5 mmol_Omim+_ g^−1^ IL: OmimPF_6_ T: 35 °C AC: CAPSUPER (highest A_BET_)	Lemus et al., 2012 [24]
BmimBr OmimBr DdmimBr Bis–DdmimBr BmpyrrBr OmpyrrBr BpyBr OpyBr	2 commercial ACs (China and fabric AC (900-20 from Kuraray))	Washed with HCl and Milli-Q water and dried (110 °C)	A_BET_ = 1044–1910 m^2^ g^−1^ pH_PZC_ = 8–8.7	C_0_ = 0–10 mM W = 2000 mg L^−1^ T = 25–55 °C pH = 7	q_L_ = 0.23–1.4	q_L_ = 1.4 mmol_Ddmim+_ g^−1^ IL: OmimPF_6_ T: 35 °C AC: fabric AC (highest A_BET_ and micropore area)	Hassan et al., 2014 [44]
BmimCl Bmim MeSO_3_ BmimOTf BmimNTf_2_ BmpyNTf_2_	Commercial AC (Merck)	No treatment	A_BET_ = 927 m^2^ g^−1^	C_0_ = 100–500 mg L^−1^ W = 250 mg L^−1^ T = 35 °C Neutral pH Na_2_SO_4_ = 0–1.76 mM	q_L_ = 0.13–1.41	q_L_ = 1.41 mmol_Bmpy+_ g^−1^ IL: BmpyNTf_2_ T: 35 °C AC: Merck	Neves et al., 2014 [45]
EmimCl HmimCl OmimCl BmimTFA BmimBF_4_ BmimOTf BmimPF_6_ BmimNTf_2_	Cellulose	Hydrothermal carbonization (250 °C, 10 h) and KOH activation (400–800 °C)	A_BET_ = 289–838 m^2^ g^−1^	C_0_ = 0–5 mM W = 2500 mg L^−1^ T = 25 °C Neutral pH	q_L_ = 0.43–0.95	q_L_ = 0.95 mmol_Omim+_ g^−1^ IL: OmimCl T: 25 °C AC: Cellulose AC	Wang et al., 2015 [46]
EmimBF_4_ EmimPF_6_ EmimNTf_2_ BmimBF_4_ PmimBF_4_	Straw ashes Wood	Straw ashes: washed with HCl and water Wood: slow pyrolysis (700 °C, 6 h)	A_BET_ = 465–1268 m^2^ g^−1^	C_0_ = 0.5–3 mM W = 4000 mg L^−1^ T = 25 °C Neutral pH	q_L_ = 0.15–0.40	q_L_ = 0.4 mmol_Pmim+_ g^−1^ IL: PmimBF_4_ T: 25 °C AC: Cellulose AC	Shi et al., 2016 [47]
BmimCl	Peanut shell Corn stalk Wheat straw	Pyrolysis (700 °C, 2 h), KOH activation (700 °C, 2 h) and washed with HCl and water. Oxidation with (NH_4_)_2_S_2_O_8_ at room temperature	A_BET_ = 1283–1347 m^2^ g^−1^	C_0_ = 0–8 mM W = 1200 mg L^−1^ T = 25 °C pH: 4.0–10.0	q_L_ = 0.61–0.85	q_L_ = 0.85 mmol_Bmim+_ g^−1^ IL: BmimCl T: 25 °C AC: Peanut shell AC oxidized with (NH_4_)_2_S_2_O_8_	Yu et al., 2016a [48]
BmimCl	Bamboo	Pyrolysis (700 °C, 2 h), KOH activation (700 °C, 2 h) and washed with HCl and water. Oxidation with (NH_4_)_2_S_2_O_8_ at room temperature	A_BET_ = 1137–1195 m^2^ g^−1^	C_0_ = 0–8 mM W = 1200 mg L^−1^ T = 25 °C pH: 4.0–10.0	q_L_ = 0.51–0.56	q_L_ = 0.56 mmol_Bmim+_ g^−1^ IL: BmimCl T: 25 °C AC: Bamboo Ac oxidized with (NH_4_)_2_S_2_O_8_	Yu et al., 2016b [49]
BmimCl HmimCl OmimCl	Cambridge filter Japan AC	Introduced in an adsorption column	A_BET_ = 1300 m^2^ g^−1^	C_0_ = 2.74 mM W = 1200 mg L^−1^ T = 30–50 °C Neutral pH	q_L_ = 0.40–0.70	q_L_ = 0.70 mmol_Omim+_ g^−1^ IL: OmimCl T: 30 °C AC: Cambridge filter Japan AC	Ushiki et al., 2017 [50]
BmimCl OmimCl HdmimCl	Ordered microporous carbon ZTC, ordered mesoporous carbon CMK–3 and 2 commercial AC (coconut-shell-derived activated carbon (HuaJing Co.), Filtrasorb-300 (Calgon Carbon Co.))	Ordered microporous carbon: Y zeolite with chemical carbon deposition of acetylene (550 °C, 5 h) and pyrolysis (850 °C, 6 h). Ordered mesoporous carbon: SBA-15 mixed with sucrose, pyrolysis (850 °C, 6 h). Both materials were washed with HF and water to remove the zeolite.	A_BET_ = 631–1610 m^2^ g^−1^	C_0_ = 0–1.25 mM W = 250–750 mg L^−1^ T = 20 °C pH: 3.5–10.0	q_L_ = 0.18–3.40	q_L_ = 3.40 mmol_Hdmim+_ g^−1^ IL: HdmimCl T: 20 °C AC: Ordered microporous carbon ZTC	Zhang et al., 2018a [51]
BmimCl	Ordered mesoporous carbon CMK–3 and AC (Filtrasorb-300, Calgon)	Ordered mesoporous carbon: SBA-15 mixed with sucrose, pyrolysis (850 °C, 5 h) and washed with HF and water to remove the silica. Both materials were oxidized using HNO_3_	A_BET_ = 762–1078 m^2^ g^−1^	C_0_ = 0–0.98 mM W = 250 mg L^−1^ T = 25–45 °C pH: 2.0–10.0	q_L_ = 0.23–0.87	q_L_ = 0.87 mmol_Bmim+_ g^−1^ IL: BmimCl T: 25 °C AC: OMC oxidized with 6 M HNO_3_	Zhang et al., 2018b [52]

**Table 2:** Characterization of the activated carbons.

Material	Adsorption-Desorption N_2_ Isotherm			Elemental Analysis					Ash (%)	TPD		pH_slurry_
	A_BET_ (m^2^/g)	V_meso_ (cm^3^/g)	V_micro_ (cm^3^/g)	C (%)	H (%)	N (%)	S (%)	O * (%)		CO (µmol/g)	CO_2_ (µmol/g)	
**Merck**	882	0.040	0.368	89.54	0.67	0.53	0.73	3.79	4.73	544	322	7.7
**Chem**	1392	0.170	0.549	84.30	0.25	1.30	0.09	9.26	4.80	1157	542	6.2
**GXS**	1065	0.270	0.391	86.70	1.00	0.58	0.78	2.53	8.41	785	350	5.0
**N_2_/KOH**	1304	0.029	0.605	70.32	0.90	0.22	0.03	12.93	15.6	867	491	8.0
**HTC/KOH**	815	0.025	0.354	74.33	0.93	0.28	0.14	8.16	16.3	725	441	9.0

* Oxygen content calculated by difference: O = 100 − %C − %H − %N − %S − %Ash.

**Table 3:** Langmuir parameters for ILs adsorption on the ACs.

IL	AC	pH	q_L_ Cation (mmol g^−1^)	k_L_ Cation (L mmol^−1^)	r^2^	q_L_ Anion (mmol g^–1^)	k_L_ Anion (L mmol^–1^)	r^2^	q_L_ Cation/q_L_ Anion	K_OW_ *
**BmimCl**	Merck	Neutral (7.3)	0.293 ± 0.004	8.86 ± 0.89	0.995	0.242 ± 0.020	4.75 ± 2.10	0.882	1.21	0.0012
**BmimAc**	Merck	Neutral (7.5)	0.861 ± 0.089	0.902 ± 0.291	0.946	0.973 ± 0.069	0.597 ± 0.110	0.986	0.88	0.9126
**BmimMeSO_4_**	Merck	Neutral (7.5)	0.707 ± 0.035	2.97 ± 0.67	0.963	0.725 ± 0.026	1.64 ± 0.230	0.989	0.98	0.0085
**BmimNTf_2_**	Merck	Neutral (7.6)	1.08 ± 0.033	13.2 ± 2.79	0.975	1.05 ± 0.037	8.01 ± 1.72	0.971	1.03	2754.2
**BmimNTf_2_**	Chem	Neutral (7.6)	0.990 ± 0.013	5.81 ± 0.43	0.996	1.01 ± 0.02	3.03 ± 0.30	0.993	0.98	
**BmimNTf_2_**	Norit	Neutral (7.6)	0.850 ± 0.021	4.77 ± 0.62	0.988	0.912 ± 0.033	2.02 ± 0.30	0.985	0.93	
**BmimNTf_2_**	Merck	4	1.74 ± 0.09	2.57 ± 0.57	0.971	1.87 ± 0.11	1.39 ± 0.29	0.976	0.93	
**BmimNTf_2_**	Merck	8	0.851 ± 0.027	12.5 ± 2.52	0.975	0.893 ± 0.032	2.54 ± 0.40	0.983	0.95	
**BmimNTf_2_**	HTC/KOH	Neutral (7.6)	1.31 ± 0.05	4.64 ± 1.02	0.972	1.39 ± 0.080	2.00 ± 0.46	0.969	0.94	
**BmimNTf_2_**	N_2_/KOH	Neutral (7.6)	2.36 ± 0.07	13.1 ± 2.7	0.098	2.51 ± 0.05	4.08 ± 0.41	0.993	0.94	
**BmimNTf_2_**	N_2_/KOH	4	3.36 ± 0.18	6.13 ± 1.88	0.966	3.53 ± 0.14	1.92 ± 0.28	0.989	0.95	
**EmimNTf_2_**	N_2_/KOH	Neutral (7.6)	1.55 ± 0.025	25.2 ± 2.86	0.993	1.60 ± 0.05	7.88 ± 1.57	0.977	0.97	323.59
**HmimNTf_2_**	N_2_/KOH	Neutral (7.7)	2.77 ± 0.14	5.34 ± 1.17	0.961	2.89 ± 0.14	3.06 ± 0.60	0.992	0.96	16,762
**OmimNTf_2_**	N_2_/KOH	Neutral (7.7)	3.20 ± 0.15	5.46 ± 0.60	0.995	2.91 ± 0.37	7.28 ± 2.48	0.962	1.10	809,717

* Kow values have been estimated by COSMO-RS.

**Table 4:** ILs used in this study.

IL	Abbreviation	Purity (%)
1–Ethyl–3–methylimidazolium bis(trifluoromethanesulfonyl)imide	EmimNTf_2_	99
1–Butyl–3–methylimidazolium chloride	BmimCl	98
1–Butyl–3–methylimidazolium acetate	BmimAc	>95
1–Butyl–3–methylimidazolium methylsulfate	BmimMeSO_4_	>95
1–Butyl–3–methylimidazolium bis(trifluoromethanesulfonyl)imide	BmimNTf_2_	99
1–Hexyl–3–methylimidazolium bis(trifluoromethanesulfonyl)imide	HmimNTf_2_	99
3–Methyl–1–octylimidazolium bis(trifluoromethanesulfonyl)imide	OmimNTf_2_	99

## Data Availability

The data presented in this study are available from the corresponding author upon request.

## Abbreviations

The following IL abbreviations are used in this manuscript:

IL	Abbreviation
Bis–DdmimBr	Dodecane–diyl–bis(methylimidazolium bromide)
BmimBF_4_	1–Butyl–3–methylimidazolium tetrafluoroborate
BmimBr	1–Butyl–3–methylimidazolium bromide
BmimCl	1–Butyl–3–methylimidazolium chloride
BmimMeSO_3_	1–Butyl–3–methylimidazolium methylsulfonate
BmimNTf_2_	1–Butyl–3–methylimidazolium bis(trifluoromethanesulfonimide)
BmimOTf	1–Butyl–3–methylimidazolium trifluoromethanesulfonate
BmimPF_6_	1–Butyl–3–methylimidazolium hexafluoroborate
BmimTFA	1–Butyl–3–methylimidazolium trifluoroacetate
BmpyNTf_2_	1–Butyl–3–methylpyridinium bis(trifluoromethanesulfonimide)
BmpyrrBr	1–Butyl–1–methylpyrrolidinium bromide
BpyBr	1–Butylpyridinium bromide
C_2_NH_2_Emim	1–Aminoethyl–3–methylimidazolium
DdmimBF_4_	1–Dodecyl–3–methylimidazolium tetrafluoroborate
DdmimBr	1–Dodecyl–3–methylimidazolium bromide
DdmimCl	1–Dodecyl–3–methylimidazolium chloride
DdmimPF_6_	1–Dodecyl–3–methylimidazolium hexafluoroborate
DmimBF_4_	1–Decyl–3–methylimidazolium tetrafluoroborate
DmimCl	1–Decyl–3–methylimidazolium chloride
DmimPF_6_	1–Decyl–3–methylimidazolium hexafluoroborate
EmimBF_4_	1–Ethyl–3–methylimidazolium tetrafluoroborate
EmimCl	1–Ethyl–3–methylimidazolium chloride
EmimNTf_2_	1–Ethyl–3–methylimidazolium bis(trifluoromethanesulfonimide)
EmimPF_6_	1–Ethyl–3–methylimidazolium hexafluoroborate
HdmimCl	1–Methyl–3–hexadecylimidazolium chloride
HmimBF_4_	1–Hexyl–3–methylimidazolium tetrafluoroborate
HmimCl	1–Hexyl–3–methylimidazolium chloride
HmimNTf_2_	1–Hexyl–3–methylimidazolium bis(trifluoromethanesulfonimide)
HmimPF_6_	1–Hexyl–3–methylimidazolium hexafluorophosphate
HOEmim	1–Hydroxyethyl–3–methylimidazolium
MmimCl	1,3–Dimethylimidazolium chloride
OmimBF_4_	1–Methyl–3–octalimidazolium tetrafluoroborate
OmimBr	1–Methyl–3–octalimidazolium bromide
OmimCl	1–Methyl–3–octalimidazolium chloride
OmimPF_6_	1–Methyl–3–octalimidazolium hexafluoroborate
OmpyrrBr	1–Octyl–1–methylpyrrolidinium bromide
OpyBr	1–Octylpyridinium bromide
PmimBF_4_	1–Pentyl–3–methylimidazolium tetrafluoroborate
PrmimNTf_2_	1–Methyl–3–propilimidazolium bis(trifluoromethanesulfonimide)
TdmimCl	1–Methyl–3–tetradecylimidazolium chloride
